# Description of an integrated management system for invasive mosquitoes at entry-exit ports in Zhejiang, China

**DOI:** 10.1186/s13071-019-3709-z

**Published:** 2019-09-18

**Authors:** Tianci Yang, Yili Lin, Cheng Li, Guojun Xie, Jun Qian, Jianmin Yang, Xiang Ma, Limin Wang, Runzi Qi, Bin Yu, Wei Zheng, Zhonghua Wu, Xiaobin Zhang, Xuechun Cao, Jie Li

**Affiliations:** 1 Hangzhou Customs District, Hangzhou, 310012 Zhejiang People’s Republic of China; 2 Zhejiang Academy of Agricultural Sciences, Hangzhou, 310021 Zhejiang People’s Republic of China; 3 Ningbo Customs District, Ningbo, 315012 Zhejiang People’s Republic of China

**Keywords:** Invasive mosquito, Quarantine, Surveillance, Online identification, Lucid

## Abstract

**Background:**

As mosquitoes are one of the most harmful creatures in the world, recent high-frequency interceptions of invasive mosquito species have emphasized the need to enhance the biological security of the Zhejiang Province in China. As such, an integrated management system should be implemented to monitor the vectors of mosquito-borne diseases during data digitization and the processing of permanent E-forms and provide an online one-stop identification service.

**Methods:**

This system is a semi-open network built on the latest Microsoft.NET Framework, Active Server Page.NET (ASP.NET) and Internet Information Services (IIS) for the Windows 2000 service as a basic infrastructure platform. This creates a physical separation between the data input as the back-page intranet and the online automated Lucid identification as the front-page internet through the digital interchange platform and security firewall.

**Results:**

This system mainly comprises three core modules: automated statistical analysis of operational data, online vector identification and digital specimen storage management, in addition to accessory modules. The joint analysis of invasive and native data collected between 2011 and 2017 at 14 surveillance points in the Zhejiang Province, excluding Ningbo Port, provided insights into the geographical differences in species abundance and the dynamic nature of seasonal interception within the statistical analysis module. Most importantly, multi-access keys to mosquitoes based on Lucid software were loaded in the module for vector identification. Subscribers can utilize this procedure for the online identification of 2 subfamilies, 10 genera and 33 mosquitoes by selecting any typical morphological feature in the classification system that matches the current images at hand.

**Conclusions:**

Our report suggests that this system can enhance the ability to master the basic information on invasive mosquitoes and satisfy the increasing requirements for public health safety in the integrated management of vector-borne diseases.

## Background

Although a mere 2–10 mm in length, the mosquito ranks as one of the most dangerous creatures on the list of “100 of the World’s Worst Invasive Alien Species” [1, 2]. Certain harmful mosquitoes are the primary vectors of malaria, yellow fever, dengue fever, chikungunya, West Nile, Zika and Japanese encephalitis, and lymphatic filariasis. Over 80% of the global population lives in areas at risk from at least one major vector-borne disease [3].

The recent high frequency of interceptions of exotic mosquito species has emphasized the need to enhance the biological security of the Zhejiang Province, located in the southeastern coastal region of China. The interception frequency of invasive mosquitoes has rapidly increased since 2004, including the yellow fever vector *Aedes aegypti* (2010; Zhoushan), malaria vector *Anopheles jeyporiensis* (2011; Quzhou) and Japanese encephalitis vector *Culex gelidus* (2016; Hangzhou), which have been captured from international aircrafts, ships, containers and commodities at outbound or inbound ports in Zhejiang.

Moreover, inbound individuals infected with the chikungunya virus (2012 and 2019 in Hangzhou) and Zika virus (2016 in Hangzhou and Yiwu) were detected when they returned to mainland China. Furthermore, three significant outbreaks of dengue occurred in 2004, 2009 and 2017, respectively, in Zhejiang Province [4].

Emerging or reemerging mosquito-borne diseases represent a substantial burden to global economies and public health [5]. Thus, an integrated management system for invasive mosquitoes should be implemented to monitor the vectors of mosquito-borne diseases to promptly determine the mosquito abundance, species identity, invasion frequency, spatial distribution and seasonal variation [6].

## Methods

### Technological framework of the integrated mosquito system

The three-tier browser/server architecture of the integrated management system for invasive mosquitoes was based on the latest Microsoft.NET Framework, using fundamental logic processing as a basic infrastructure platform. Active Server Page.NET (ASP.NET) and Internet Information Services (IIS) for the Windows® Server were utilized to create an interactive foreground website and background support operating platform, respectively. To support the transactional database, MySQL 5.6 was employed for fast access to data under high-throughput conditions of stability, security and scalability on this operating platform.

**Fig. 1:**
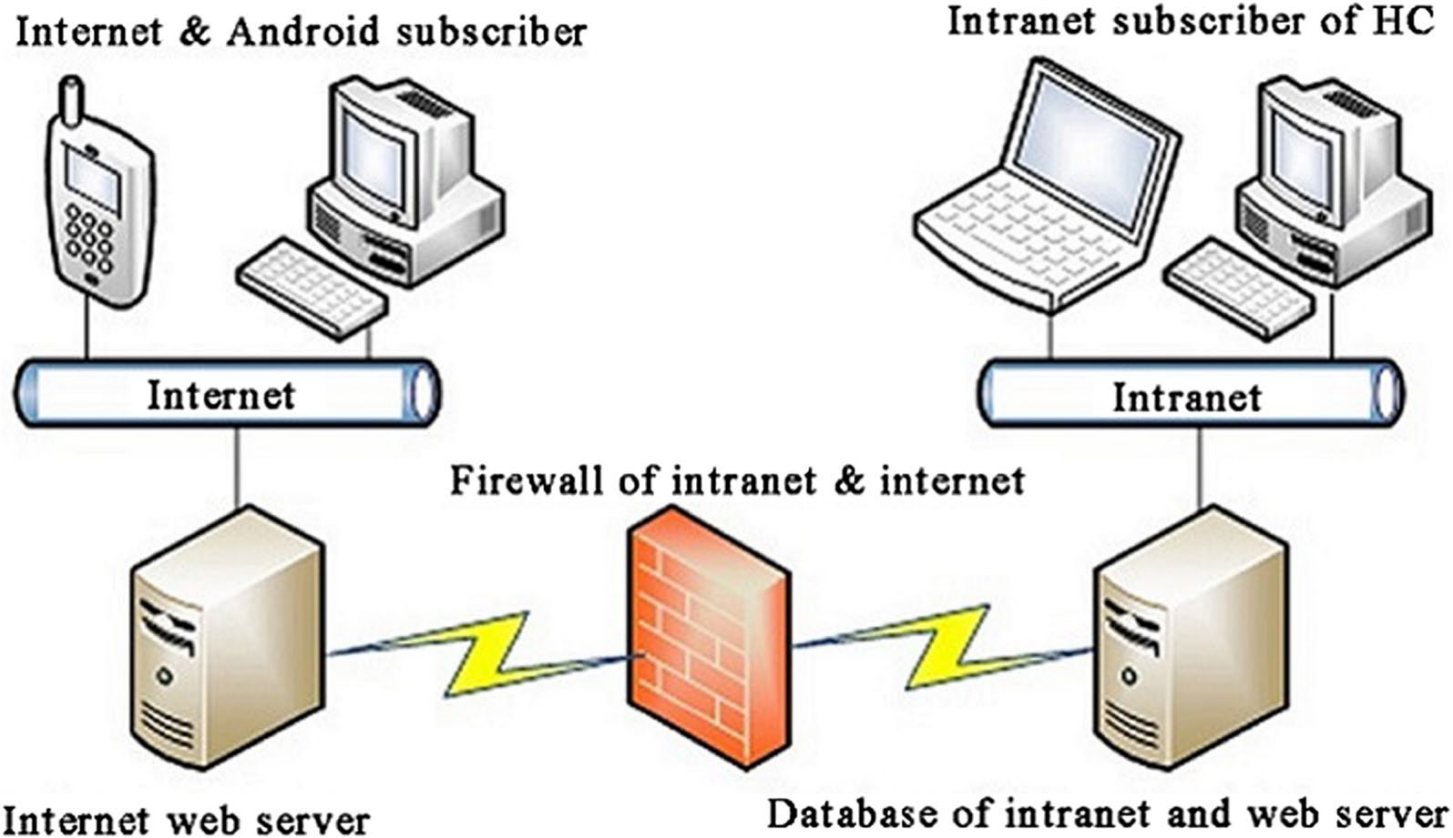
Framework of integrated management system for invasive mosquitoes at Zhejiang ports, China. *Abbreviation*: HC, Hangzhou Customs

The proposed integrated management system for invasive mosquitoes is a semi-open network platform, which creates a physical separation between the data input as the back-page intranet and the online automated identification as the front-page internet, through a digital interchange platform and security firewall (Fig. 1). A subscriber from a surveillance point submits the application tasks, handles data entry, fills in relevant information and uploads images or multimedia videos through the intranet network. Then, the technical staff in the laboratory complete morphological identification, pathogen detection, DNA barcoding and electronic reports according to the requirements of the above tasks. Next, the subscriber that submitted the application task checks the results of the final laboratory detection reports and downloads the electronic documents at any time. Moreover, the front-page internet interface allows the subscriber to access an introduction to medical vectors, details from popular science, achievements in scientific research, interactive computer identification of mosquitoes and online technical support with entomologist experts.

### Functional modules of the integrated management system

The integrated management system is mainly composed of three core modules: automated statistical analysis of operational data, online vector identification and digital specimen storage management in the process of invasive mosquito surveillance, as well as some accessory modules.

In the module for automated statistical analysis of the operational data, the contents are submitted to the management system, including steps of sample registration and reception, and morphological and molecular identification of exotic mosquitoes. These data are then analyzed to automate the monthly statistical sheet, charts for population composition, seasonal variations in exotic mosquitoes and reports of inbound individuals with mosquito-borne diseases, in comparison with the local surveillance of mosquitoes and mosquito-borne diseases.

In the online vector identification module, details on common important mosquitoes are stored, along with those of midges, ticks, fleas and other species, epidemic situations and the option to utilize a Lucid multi-access key to confirm the identification of mosquitoes.

In the digital specimen storage management module, once a task is terminated, basic information on the species is stored in the module, including records of specimen storage, storage locations, new specimens or specimens damaged in the course of usage.

### Data acquisition of mosquitoes and vector-borne diseases

**Fig. 2:**
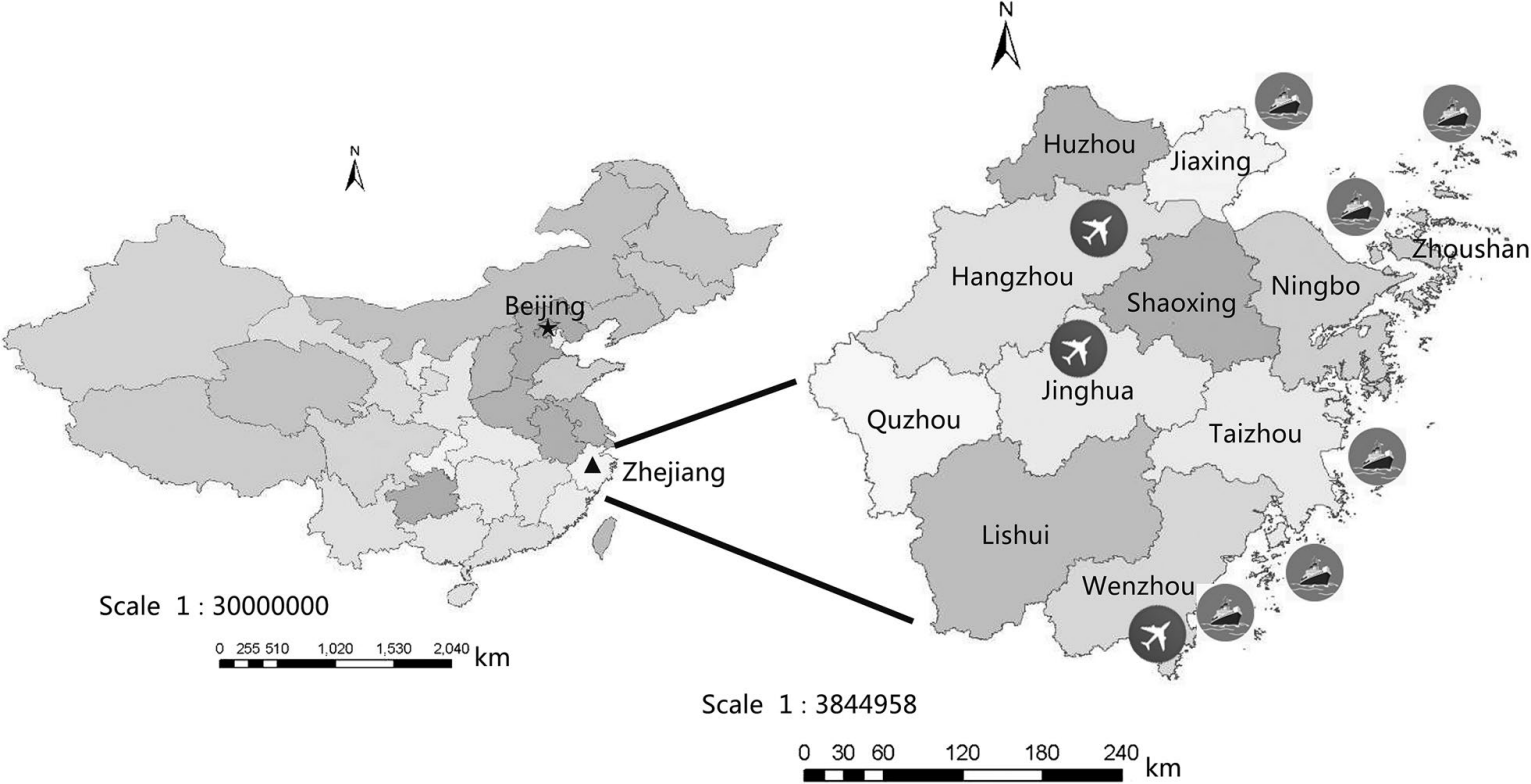
Geographical distribution of surveillance points in Zhejiang Province, China (obtained from the National Platform for Common Geospatial Information Services)

All data on invasive mosquitoes were obtained from 14 surveillance points in Zhejiang Province excluding Ningbo Port (Fig. 2). These included three airports in Hangzhou, Wenzhou and Yiwu; four seaports in Wenzhou, Zhoushan (Jintang and Shengsi), Taizhou (Haimen and Damaiyu) and Jiaxing; and the Hangzhou mail cover checkpoint.

The invasive mosquitoes were sampled from incoming quarantined aircrafts, ships, containers (cargoes and cars) and commodities using mechanical aspiration for adults and dipping for larvae. Local mosquitoes were also sampled from sentinel sites designated at the 14 surveillance points using carbon-dioxide-baited mosquito traps (Shenlei Co. Ltd., Shanghai, China), mosquito light traps (Kongfu Dude Co. Ltd., Wuhan, China), mosquito nets (BioDiVector, WLi Enviro Protection, Suzhou, China) or mosquito oviposition traps (Heli Kechuang Co. Ltd., Beijing, China).

Moreover, all details of reports of inbound individuals with mosquito-borne diseases at Zhejiang ports were collected for the final detection reports of our unit, and other resources were directly reprinted from the global infectious diseases (GID) information provided by the General Administration of Customs, P. R. China.

### Tools for online interactive mosquito identification

Tools for online interactive mosquito identification were developed based on Lucid software (Identic Pty Ltd., Queensland, Australia; http://www.lucidcentral.com). Lucid Professional v.3.51 was utilized to link multiple typical features of the head, thorax and abdomen [2, 7–11] to the respective entities of mosquito genera, listed in a group of important entries for Zhejiang invasive and native mosquitoes. Fact Sheet Fusion (Identic Pty Ltd) facilitated the rapid generation of the morphological characteristics of each species, with additional images in hypertext markup language (HTML). The diagnostic keys to mosquitoes were deployed using the links between features and categories over the web-based management system in Lucid3 Builder.

Some images cited in the keys were downloaded from the website of the Walter Reed Biosystematics Unit (WRBU, http://wrbu.org) [12], the Mosquitoes of Europe [13] software and the latest books and references [14, 15].

## Results

### Main interface and operational procedures of the system

The newly integrated management system for mosquitoes is divided into two components: a back-page intranet (http://10.73.10.185) and front-page internet (http://60.191.36.206:9090). When subscribers from the surveillance points are authorized to log into the intranet, they submit the online task application and mission contents by clicking one of the icons corresponding to vector surveillance, sample identification detection, data for statistical analysis, specimen management or internet publishing from the left menu.

For samples of mosquitoes captured at different ports, a subscriber must fill out the online E-form task application sheet, including the types of transportation, entry-exit countries, original production sites and insect quantities, and select from alternative options regarding morphological identification, pathogen detection, DNA barcoding and others. As soon as the technical staff in the laboratory receive the instructions from the intranet and the sample material, they immediately initiate ongoing detection, scan the final documents with the seal and convert these to portable document format (PDF) reports. Then, the subscriber can download the final versions of the online PDF reports and even check the current consumption or storage of samples.

### Statistical analysis of operational data module

**Fig. 3:**
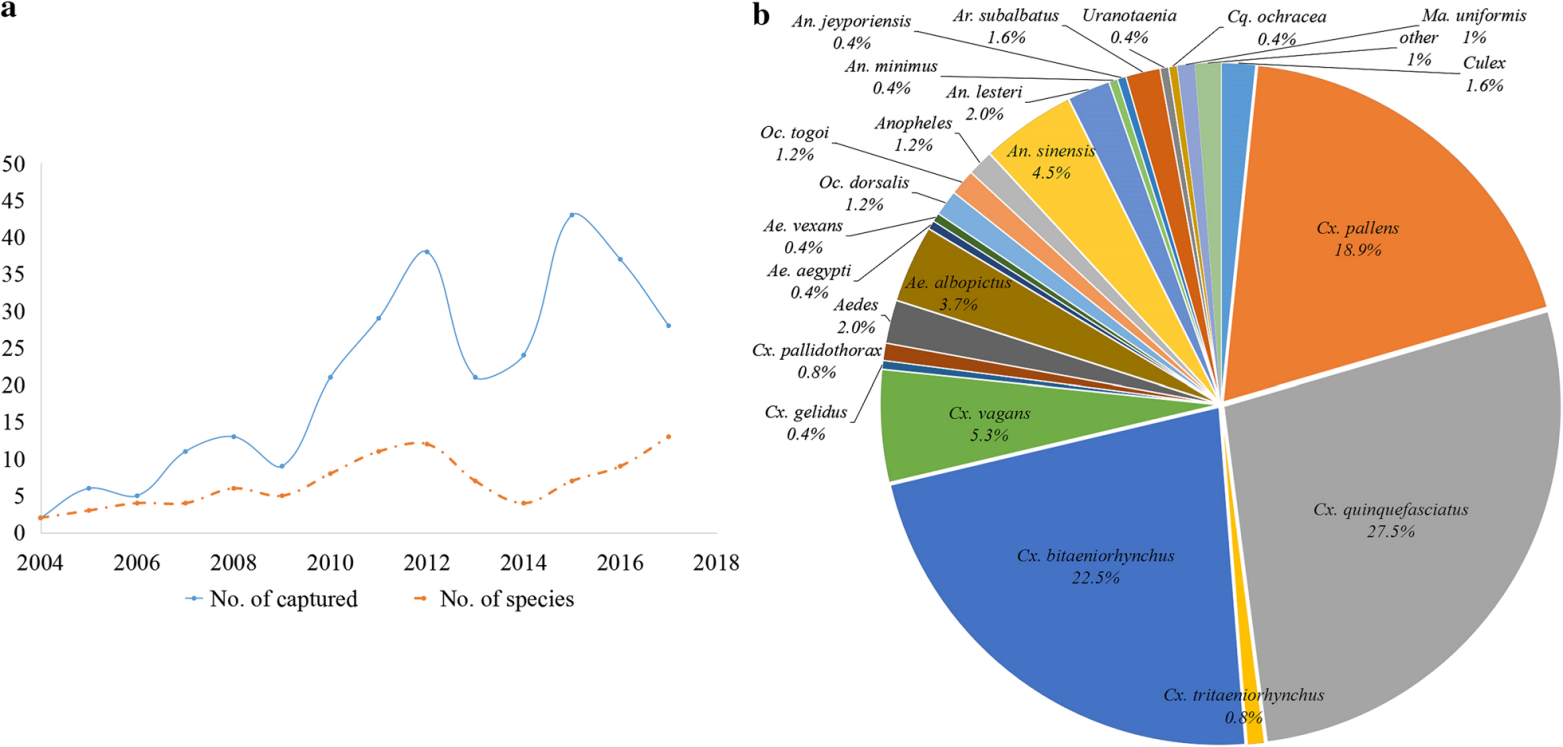
Annual trends and species abundance of captured mosquitoes from 2011 to 2017 in the Zhejiang Province, China. **a** Dynamic variation of captured invasive mosquitoes. **b** Pie chart of mosquito species composition

For the automated statistical analysis of the operational data module, in addition to the capture of invasive mosquitoes, all the data on native mosquito species collected from 2011 to 2017 at Zhejiang ports during routine surveillance will be logged in the integrated management system. A subscriber can look through the actual information on vector composition, capture frequency, species abundance of exotic mosquitoes, invasion routes and countries of origin, and compare the data with results from the local routine surveillance of mosquitoes, while automatically producing a variety of line graphs, pie charts and histograms on the backstage computer (Fig. 3).

### Module of online vector identification

In the online vector identification module, a subscriber can utilize the internet interface to browse through details of medical entomology, spatial distributions, biological characteristics, life-cycles and the medical importance of mosquitoes, along with midges, ticks, fleas and other species. A subscriber may also peruse the guide to vector-borne diseases, a presentation of academic publications, achievements in scientific research and popular science education.

**Fig. 4:**
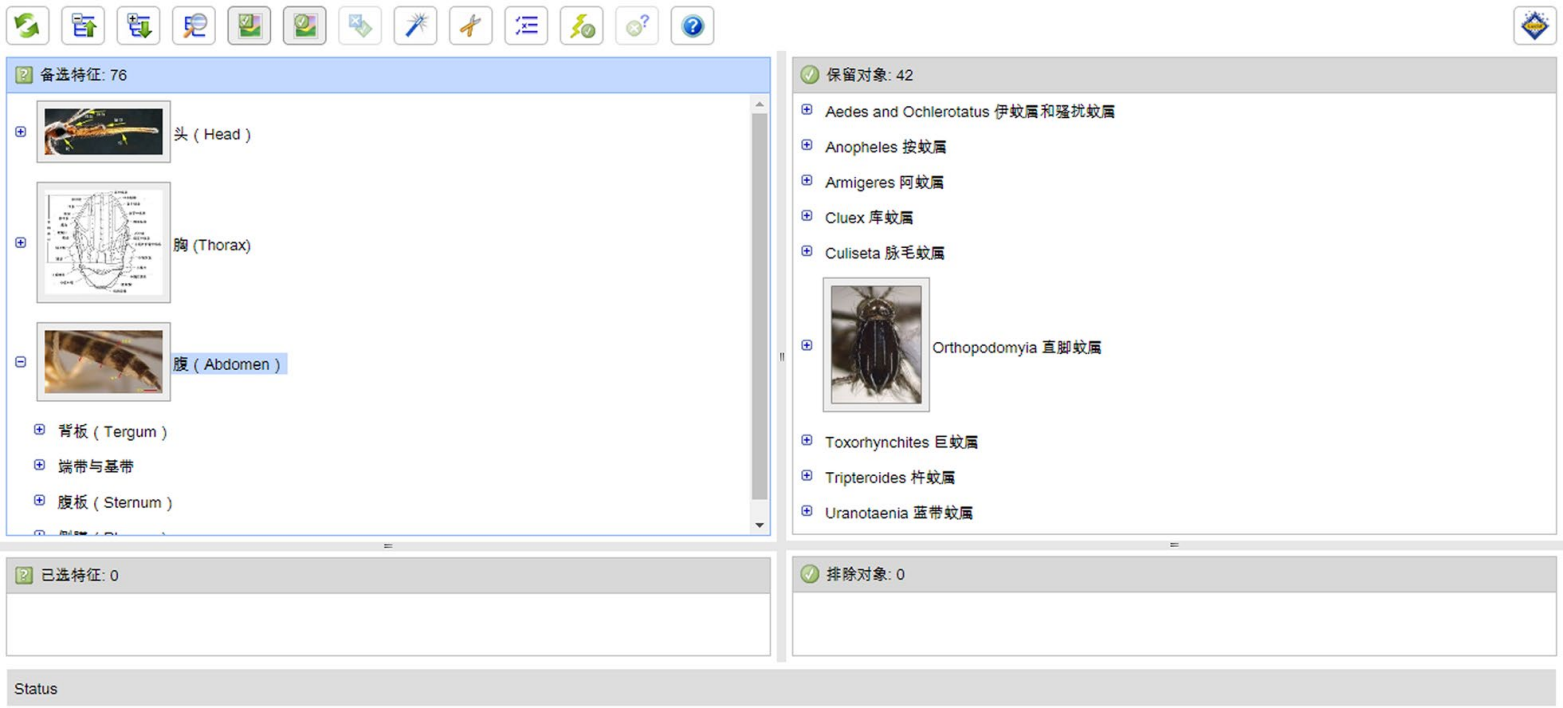
Tools for online classification and taxonomy of mosquito identification keys using Lucid on the front-page internet (http://www.livefarm.cn/lucid/mosquito)

Most importantly, multi-access keys to mosquitoes based on Lucid software are available in this module. A subscriber can click on a typical feature from a total of 76 alternative features in the entries of the left column, which are divided into the three distinct body regions of the head, thorax and abdomen, and cross-check the images that they retrieve (Fig. 4). Once a selected feature is displayed at the lower-left corner of the identification keys, the backstage system will automatically match the selected feature with one of the potential retrieval objects for 33 mosquito species from 10 genera and 2 subfamilies, showing the single matched object in the top-right corner and the discarded objects in the lower-right corner. Normally, only one step is required to end the online identification procedure. If more than one object remains in the top-right corner, then the subscriber can continue to pick out additional features from those that remain for matching until the completion of the identification of the mosquitoes. If the final task cannot be completed, then it is likely that the specimen is not recorded in the identification keys, which will be further upgraded in the future.

**Fig. 5:**
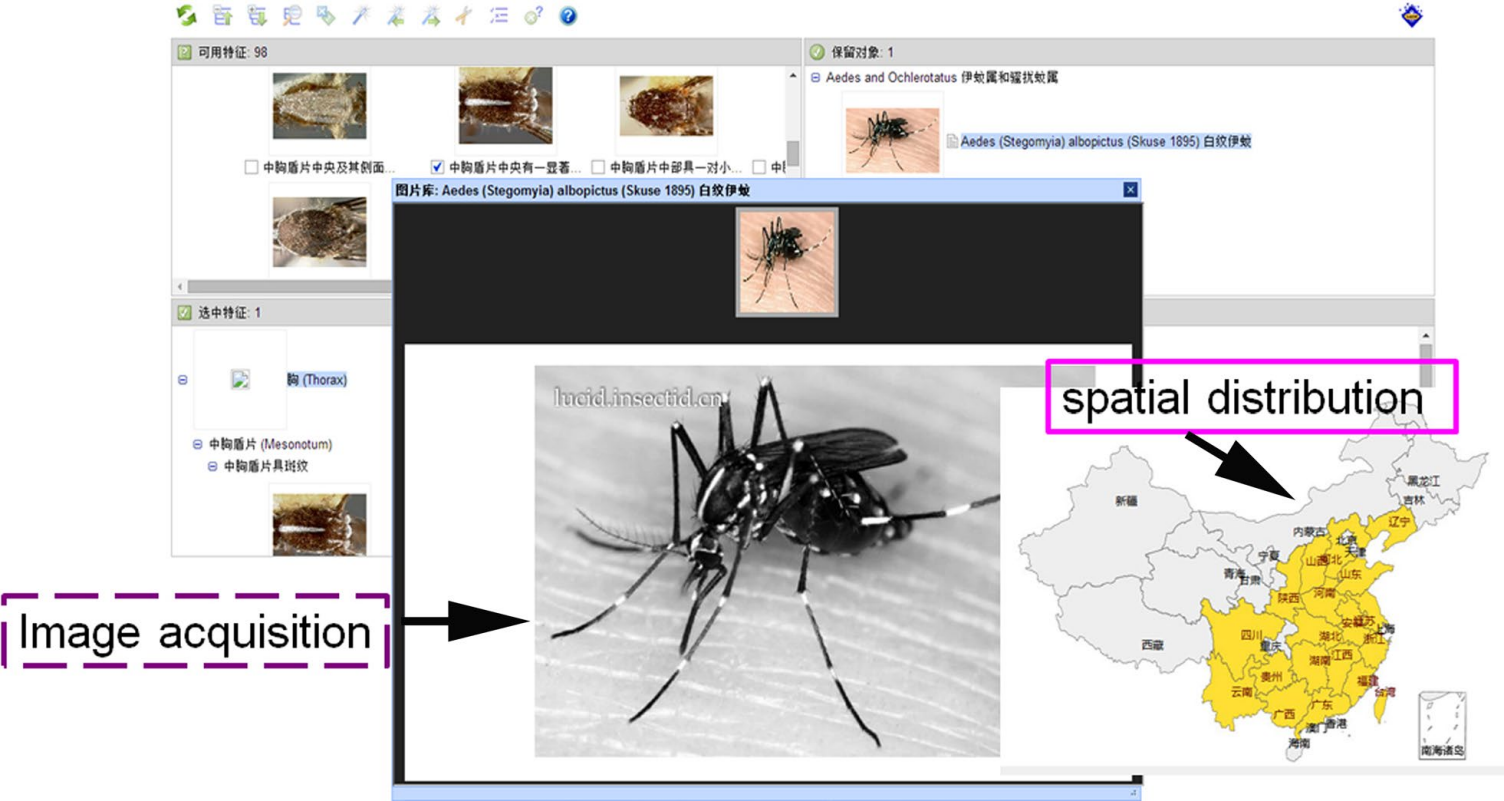
Related supplemental information on exotic mosquitoes obtained from the identification keys on the front-page internet

The geographical distribution of mosquito-borne diseases or vectors on the website or mobile Android application (App) will be visualized using an application programming interface (API) provided by Baidu Maps (Fig. 5). When the procedure is finished, the subscriber can continue to click on the images or text media icons of the remaining object in the top-right corner. These icons will be further magnified to demonstrate some real-time details of taxonomically important images, biology, medical importance and the spatial distribution on the screen of the desktop or notebook computer. At the same time, this system can provide precaution levels for important blood-sucking taxa, such as mosquitoes, midges, ticks and fleas.

## Discussion

Zhejiang should be listed as at moderate risk of mosquito-borne diseases, as there were three outbreaks of dengue in 2004, 2009 and 2017, respectively [4], and some reports of infections in inbound individuals testing positive for chikungunya (2012 and 2019), Zika (2016) and malaria (after 2011).

Because of the limitations of development costs, the functional layout of this integrated management system may not satisfy the demands of modern inspection and quarantine, and it is likely to remain stable on the Windows XP, 7 or 10 operating systems. The program will probably not run smoothly on the 360, Internet Explorer or Google Chrome browsers. All data on mosquito-borne diseases collected and recorded in recent years are insufficient in this management system, and the functionality of the automated statistical analysis needs to be tested during the vector data transmission process.

Moreover, the images cited in the Lucid interactive mosquito identification keys need to be authorized from official websites for use domestically and internationally, including the WRBU or IIKCulicoide (http://www.iikculicoides.net) [12, 14]. Although we recorded some images of the captured invasive vectors as our own intellectual property, some unsatisfactory flaws remain in the resolution of images during the photomicrography and digital graphics processing.

In view of the indicators for implementation of the Global Vector Control Response (GVCR) 2017–2030 of the World Health Organization [3], which outlines new and broad principles and approaches to vector control that are applicable to all vector-borne diseases, we need to build a comprehensive information management system for mosquito-borne diseases and vectors at Zhejiang ports. In particular, it is necessary to develop apps for important blood-sucking species (ticks, fleas, midges and blackflies); promote the construction of infrastructure in terms of photomicrography, morphometrics and a support vector machine algorithm for species identification [15]; and pursue special funds for the secondary exploitation of this system to expand the database of vectors and satisfy the grounding requirements for public health and health education of young adults [16].

## Conclusions

Our report suggests that the proposed system can enhance the ability to master basic information on invasive mosquitoes and satisfy the grounding requirements for public health safety during the integrated management of vector-borne diseases.

## Data Availability

All data and materials are available in this published article.

## Abbreviations

API: application programming interface
app: application
ASP: active server page
GID: global infectious diseases
GVCR: Global Vector Control Response
HC: Hangzhou Customs
HTML: hypertext markup language
IIS: internet information services
PDF: portable document format
